# Nitrogen uptake and assimilation in proliferating embryogenic cultures of Norway spruce—Investigating the specific role of glutamine

**DOI:** 10.1371/journal.pone.0181785

**Published:** 2017-08-24

**Authors:** Johanna Carlsson, Henrik Svennerstam, Thomas Moritz, Ulrika Egertsdotter, Ulrika Ganeteg

**Affiliations:** 1 Umeå Plant Science Centre, Department of Forest Genetics and Plant Physiology, Swedish University of Agricultural Sciences, Umeå, Sweden; 2 Svenska Skogsplantor AB, Seed Production, Lagan, Sweden; 3 Umeå Plant Science Centre, Department of Plant Physiology, Umeå University, Umeå, Sweden; 4 G.W. Woodruff School of Mechanical Engineering, Georgia Institute of Technology, Atlanta, United States of America; Youngstown State University, UNITED STATES

## Abstract

Somatic embryogenesis is an *in vitro* system employed for plant propagation and the study of embryo development. Nitrogen is essential for plant growth and development and, hence, the production of healthy embryos during somatic embryogenesis. Glutamine has been shown to increase plant biomass in many *in vitro* applications, including somatic embryogenesis. However, several aspects of nitrogen nutrition during somatic embryogenesis remain unclear. Therefore, we investigated the uptake and assimilation of nitrogen in Norway spruce pro-embryogenic masses to elucidate some of these aspects. In our study, addition of glutamine had a more positive effect on growth than inorganic nitrogen. The nitrogen uptake appeared to be regulated, with a strong preference for glutamine; 67% of the assimilated nitrogen in the free amino acid pool originated from glutamine-nitrogen. Glutamine addition also relieved the apparently limited metabolism (as evidenced by the low concentration of free amino acids) of pro-embryogenic masses grown on inorganic nitrogen only. The unusually high alanine concentration in the presence of glutamine, suggests that alanine biosynthesis was involved in alleviating these constraints. These findings inspire further studies of nitrogen nutrition during the somatic embryogenesis process; identifying the mechanism(s) that govern glutamine enhancement of pro-embryogenic masses growth is especially important in this regard.

## Introduction

Somatic embryogenesis (SE) is an *in vitro*-based technology where the zygote from one seed can be propagated into an infinite number of genetically identical plants (e.g. [1]). Since the first successful protocol of conifer SE [2–3], SE has become an important model system for studying the process of embryo development in gymnosperms (reviewed by e.g. [4–6]). The process of SE also enables rapid propagation of plants with desirable traits for commercial use and conservation purposes [7–9].

In Norway spruce (*Picea abies* L. Karst), early stage somatic embryos (pro-embryogenic masses; PEMs) form from zygotic seed embryos on ½-LP medium [10] supplemented with 2,4-diclorophenoxyacetic acid and N6-benzyladenine. The PEMs multiply to form an embryogenic culture sustained in a proliferative stage by sub-culturing every second to third week to a medium of the same composition. PEM multiplication is ended by transfer of the cultures to a DKM maturation medium [11] without plant-growth regulators, followed by complete development into mature somatic embryos on a medium containing abscisic acid [4,12]. After the maturation stage, the embryos are harvested and desiccated before germination and *ex vitro* plant acclimatization [4,7].

Nitrogen (N) metabolism is central to the growth and development of plants [13–14], and provides building blocks for the synthesis of a plethora of biomolecules, such as proteins, nucleic acids and chlorophyll. Uptake and assimilation of N are therefore key factors in plant growth, both in natural ecosystems and *in vitro* systems. Plant N uptake has typically been associated with uptake of inorganic N, i.e. nitrate (NO_3_^-^) and ammonium (NH_4_^+^), from the soil. However, during the last decades, the potential importance of organic N for plant N nutrition has also been investigated [15–16]. Studies of N uptake have shown that plants have the potential to take up amino acids (AAs), regardless of growth habitat and mycorrhizal associations [15,17–18]. Moreover, studies concerning the uptake of different N sources have shown that many conifer species have a preference for NH_4_^+^, compared with NO_3_^-^ [19–28]. More importantly, the uptake rates of AAs have been found to be similar or higher than those of NH_4_^+^ [25,27–32]. The molecular mechanism underlying AA uptake has also been identified; uptake in *Arabidopsis thaliana* is mediated mainly by the Lysine Histidine Transporter 1, Amino Acid Permease 5, Amino Acid Permease 1 and Lysine Histidine Transporter 6 [33–37].

Prior to assimilation, NO_3_^-^ is taken up from the growth substrate by nitrate transporters, or imported to the cytosol from the vacuole, and is thereafter reduced to NH_4_^+^ via a two-step reaction. The cytosolic enzyme nitrate reductase (NR) catalyses the reduction of NO_3_^-^ into nitrite (NO_2_^-^). The NO_2_^-^ is transported into the chloroplasts (green tissue) or plastids (roots), where nitrite reductase (NiR) converts NO_2_^-^ into NH_4_^+^ [38–39].

NH_4_^+^, originating from NO_3_^-^ assimilation, photorespiration or root uptake, is assimilated via the glutamine synthetase/glutamate-2-oxoglutarate aminotransferase (GS/GOGAT) pathway [40–41]. The enzymes, GS and GOGAT, produce glutamine (L-Gln) and glutamate (L-Glu) from which other AAs are synthesised [13]. The involvement of glutamate dehydrogenase (GDH) in the assimilation of NH_4_^+^ has also been suggested. However, several studies have found that GDH participates mainly in the deamination of L-Glu, i.e. NH_4_^+^ release (reviewed by [42]). The primary products of assimilated inorganic N are AAs. However, the exact fate of AAs taken up from the growth substrate remains unclear. Hypothetically, AAs are either directly incorporated into proteins or transformed into other AAs via aminotransferases [38,41]. Several studies have suggested that the metabolism of AAs depends primarily on transamination reactions, rather than on re-assimilation via GS/GOGAT of NH_4_^+^ from AA deamination [15,31,43].

In media used for plant *in vitro* applications, N is added as inorganic N (NH_4_^+^ and NO_3_^-^) and organic N (mainly AAs) [44–46]. L-Gln, one of the most common sources of organic N used in plant *in vitro* cultures, can enable or enhance growth of these cultures [47–50]. L-Gln is also commonly added during the process of conifer SE [44,51–52]. Induction and proliferation studies have been conducted on several species, e.g. silver fir (*Abies alba)* [53], white spruce (*Picea glauca* (Moench) Voss) [54] and *Picea abies* [44]. These studies showed that growth media containing L-Gln or casein hydrolysate improved initiation of new SE cultures and proliferation growth, compared with that when only inorganic N is present. Furthermore, somatic embryos of black spruce (*Picea mariana*) [52] and Fraser fir (*Abies fraseri* (Pursh) Poir.) [55] can fully mature on media where L-Gln is the sole source of N.

The incorporation of inorganic N into AA metabolism has previously been studied in the SE process of white spruce. Using isotopically labelled N, it was demonstrated that both NO_3_^-^ and NH_4_^+^ were taken up and incorporated into the metabolism; the first detected resonance in embryogenic cultures originated from the amide group of L-Gln, suggesting that NH_4_^+^ is incorporated through GS/GOGAT. Furthermore, NO_3_^-^ was assimilated, but at a slower rate than NH_4_^+^ [56]. However, there is still a paucity of information about the uptake and assimilation of N during SE in conifers.

The objective of this study was to provide additional insights into N nutrition of PEMs, with an emphasis on PEM uptake capacity of different N sources and the subsequent incorporation of these sources into AA metabolism. We hypothesised that the previously observed positive growth effect of L-Gln is due to the N species added rather than to an increase in the total N concentration. Based on previous studies showing that conifers preferentially take up AAs and NH_4_^+^, we also hypothesised that the increased growth effect results from different uptake capacities of the single N forms. We tested these hypotheses through a series of experiments addressing the importance of L-Gln for PEM growth as well as the PEM N uptake characteristics and the incorporation of each N form into the free AA pool. These experiments were performed through a combined approach of ^15^N-isotope labelling and targeted metabolite profiling of AAs via liquid chromatography-mass spectrometry (LC-MS). We found that the addition of L-Gln positively affects the growth of PEMs and is vital for maintenance of PEM cell metabolism under the conditions tested.

## Materials and methods

### Embryogenic lines and cultivation system

Norway spruce embryogenic cell lines 11:08:59 and 11:12:02 were used for the growth experiment. Cell line 11:12:02 was used for the ^15^N-isotope labelling and targeted metabolite profiling. Both cell lines were initiated at SweTree Technologies, Sweden, in 2011. Embryogenic cultures were sub-cultured biweekly on solidified ½-LP medium supplemented with 9.0 μM 2,4-diclorophenoxyacetic acid, 4 μM N6-benzyladenine, 3.1 mM L-Gln and 10 mM sucrose (10). Unless otherwise stated, 1 g fresh weight (FW) of embryogenic culture was spread out in ten patches on a 9 cm (diameter) plate with 25 mL media. The calluses growing on a single plate were all pooled into one biological replicate.

### Biomass growth of PEMs

Embryogenic cultures were transferred from the standard media (given above) to three experimental ½-LP proliferation media (PM; see Table 1), hereafter referred to as PM#1 (½-LP, 16.8 mM total-N), PM#2 (½-LP supplemented with 3.1 mM NH_4_NO_3_ (corresponding to 6.2 mM N), 23 mM total-N) and PM#3 (½-LP supplemented with 3.1 mM L-Gln (corresponding to 6.2 mM N), 23 mM total-N). PM#3 is thus the same as the standard media used for proliferation. The experimental media PM#1 and PM#2 contained the same (only inorganic) N source, but different amount of total N. Similarly, PM#2 and PM#3 contained the same amount of total N provided as different N sources (inorganic vs. inorganic+organic).

**Table 1:** Experimental proliferation media compositions with inorganic and organic N.

		Total N	NH_4_^+^-N		NO_3_^-^-N		L-Gln-N	
Medium	Treatment	mM	mM	%	mM	%	mM	%
½-LP	PM#1	16.8	3.7	22.2	13.1	77.8	-	-
½-LP + 3.1 mM NH_4_NO_3_	PM#2	23	6.8	29.6	16.2	70.4	-	-
½-LP + 3.1 mM L-Gln	PM#3	23	3.7	16.3	13.1	57.0	6.2	26.7

Experimental media, corresponding concentrations of total N and the N concentration of each N source (NH_4_^+^, NO_3_^-^ and L-Gln) provided in the media.

The embryogenic cultures used in this experiment were established and maintained on L-Gln-containing media (PM#3). Hence, residual L-Gln from the maintenance media may influence PEM growth during treatments without added L-Gln (PM#1 and PM#2). Pre-cultivating the cultures on the respective medium for two weeks minimised this influence. After the depletion period, the cultures were moved to new plates, and grown for an additional two weeks on the respective experimental medium. The FW was measured, after which the samples were frozen in liquid N and freeze-dried for 72 h (Scanvac Coolsafe freeze dryer, LaboGeneTM, Denmark). These samples were used to determine dry weight (DW) and to calculate the FW/DW ratio.

Five biological replicates (per treatment) were used for AA analysis. Samples were ground for 1 min, using a bead mill (MM400, Retsch GmbH, Germany) equipped with four 3 mm tungsten beads and set to a frequency of 20 Hz. In addition, an experiment was carried out to investigate growth of PEMs at a lower (1.5 mM) and a higher (6.2 mM) concentration of L-Gln in the proliferation media (S1 Table) in comparison to PEMs grown on PM#1. There was no depletion period before the start of the experiment, start weight was 0.9 g FW of embryogenic culture and seven biological replicates per treatment. FW and DW was determined as described above.

### Analysis of mass fraction of N, NH4+ and AA concentrations

The mass fraction of N (g N per g dry mass) of the PEMs from cell line 11:12:02 was determined using an elemental analyzer (Flash EA 2000, Thermo Fisher Scientific, Bremen, Germany). Five biological replicates (per treatment) were collected and analysed.

Freeze-dried PEM samples (4 mg) were extracted in 0.01 M HCl and AA and NH_4_^+^ concentrations were measured via the UPLC-AccQTag method (UPLC amino acid analysis system solution, www.waters.com).

### N uptake analysis

Embryogenic cultures were transferred to plates with PM#3 separately amended with ^15^ammonium chloride (^15^NH_4_Cl), potassium ^15^nitrate (K^15^NO_3_) or ^15^amine-Glutamine (^15^amine-L-Gln). The final concentration of ^15^N was 1% (K^15^NO_3_), 0.5% (^15^N-L-Gln) and 0.66% (^15^NH_4_Cl) of the respective amount of the N compound in the media. This was taken into consideration by using a multiplication factor in the calculations (see below). Each plate (25 mL of growth medium) contained 8.05 mg of N and PEMs contained a maximum amount of 3.9 mg. After two weeks, samples were collected, frozen in liquid N and stored at -20°C until further analysis. The samples were dried at 50°C for 24 h and ground, for 1½ min, into a powder using a bead mill (MM400, Retsch GmbH, Germany) set to a frequency of 25 Hz and equipped with two 5 mm beads and one 8 mm bead. The ^15^N content of the PEMs was determined using an Elemental Analyzer—Isotope Ratio Mass Spectrometer (EA-IRMS) with an isotope ratio mass spectrometer (DeltaV, Thermo Fisher Scientific, Bremen, Germany) interfaced to the element analyser (Flash EA 2000). Five biological replicates (per ^15^N treatment) were collected and analysed.

### Targeted AA analysis

The proliferation media was divided into five separate aliquots. Four aliquots were separately treated with one of the four N ^15^isotopes, ^15^NH_4_^+^ NO_3_^-^ (≥98% ^15^N), NH_4_^+ 15^NO_3_^-^ (≥98% ^15^N), ^15^amide-L-Gln (≥98% ^15^N) and ^15^amine-L-Gln (≥98% ^15^N). L-Gln contains two N atoms (one in the amine group and one in the amide group) and these were separately labelled. The amount of N-isotope compound added to the media represented 29% of NO_3_^-^ and 100% of NH_4_^+^, ^15^amide-L-Gln and ^15^amine-L-Gln. The fifth aliquot was unlabelled and used for measuring the ^15^N natural abundance. Since 29% of the NO_3_^-^ in the growth medium was labelled, a correction factor of 3.5 was used in the calculations.

The starting weight of each embryogenic culture was 300 mg (divided into three pieces) per plate (diameter: 5 cm, containing 10 mL media) and treatment. Samples were collected after two weeks of growth. The collected samples were frozen in liquid N and stored at -20°C until they were freeze-dried (Scanvac Coolsafe freeze dryer, LaboGeneTM, Denmark) for 72 h. These samples were ground for 1 min using a bead mill (MM400, Retsch GmbH, Germany) equipped with four 3 mm tungsten beads and set to a frequency of 20 Hz. Ten biological replicates (per treatment) were analysed for targeted profiling, via LC-MS analysis, of ^15^N incorporation into AAs, except for amide-L-Gln where only nine biological replicates were analysed.

Metabolite samples were extracted using a modified version of the technique described by [57]. Eight mg of freeze-dried sample and a 3 mm tungsten bead were placed in a 1.5 mL Eppendorf tube. Afterwards, 1 mL of an extraction mixture consisting of 800 μL methanol, 200 μL H_2_O and 0.25 pmol μL^-1^ norvaline was added to the tube. Samples were extracted for 3 min using a bead mill (MM400, Retsch GmbH, Germany) set to a frequency of 30 Hz s^−1^. Sample extracts were centrifuged and 200 μL of supernatant was transferred to LC vials and dried for 1½ h at 25°C, using a speed-vac concentrator (Savant Instruments, Farmingdale, NY, USA). Derivatisation of AAs in the sample extracts was performed via the Waters AccQ•Tag™ method, in accordance with the manufacturer’s protocol. The samples were analysed via LC-MS (for a more detailed description see supporting information S1 Text).

The LC-MS analysis was controlled by the MassHunter™ software package, v B.05.01 and data processing was performed using the MassHunter™ Quantitation software, v B.07.00 (Agilent Technologies Inc., Santa Clara, CA, USA).

### Calculations and statistical treatment of data

The amounts of N derived from the different N sources are reported in units of mg N in the tissue from 1 biological replicate, calculated using the formula:

$$\left((\mathrm{atom}\%\;{}^{15}N_{\mathrm{LS}}-\mathrm{atom}\%\;{}^{15}N_{\mathrm{NAT}})/100)\times (({N\%}_{\mathrm{TOT}})/100)\times \mathrm{DW}(\mathrm{mg})\times \mathrm{MF}=N_{\mathrm{LS}}\;(\mathrm{mg}\right)$$ (1)

where the subscript “LS” denotes labelled sample, “NAT” natural abundance (here 0.3663% is used for all samples) and MF denotes Multiplication Factor. The contribution of each N form is given as % of total N uptake during the labelling experiment.

The amount of N (μg) assimilated from the different N sources into AAs in the free AA pool was calculated in a stepwise manner.

Firstly, the fraction of an AA isotopologue (AreaAA^n^_LS_ divided by the sum of all isotopologue peak areas) in the labelled samples (LS) was calculated and corrected for the average natural abundance of ^15^N in non-labelled (NLS) control samples.

$$({\mathrm{Area}\;\mathrm{AA}}_{\mathrm{LS}}^{N}/{\mathrm{Area}\;\mathrm{AA}}_{\mathrm{LS}}^{0}+{\mathrm{Area}\;\mathrm{AA}}_{\mathrm{LS}}^{+1}+{\mathrm{Area}\;\mathrm{AA}}_{\mathrm{LS}}^{+2})-({\mathrm{Area}\;\mathrm{AA}}_{\mathrm{NLS}}^{N}/{\mathrm{Area}\;\mathrm{AA}}_{\mathrm{NLS}}^{0}+{\mathrm{Area}\;\mathrm{AA}}_{\mathrm{NLS}}^{+1}+{\mathrm{Area}\;\mathrm{AA}}_{\mathrm{NLS}}^{+2})=\mathrm{Fraction}\;{\mathrm{AA}}_{\mathrm{LS}}^{n}$$ (2)

where n denotes the number of ^15^N atoms in the isotopologue.

Secondly, the concentration (μmol/g DW) of each isotopologue AA^n^ was calculated as:

$$(\mathrm{Fraction}\;{\mathrm{AA}}^{n})\times {\mathrm{AA}}_{\mathrm{LS}}={\mathrm{AA}}^{n}$$ (3)

where AA_LS_ represents the concentration of the AA in the sample determined by UPLC- analysis.

Thirdly, the concentration of N (μmol /g DW) derived from the labelled N source N_LS_ was calculated as:

$${\mathrm{AA}}^{+1}+({\mathrm{AA}}^{+2}\times 2)=N_{\mathrm{LS}}$$ (4)

Lastly, the absolute amount of N (μg) derived from the labelled N source in each biological replicate was calculated as:

$$N_{\mathrm{LS}}\times {\mathrm{DW}}_{\mathrm{TOT}}\times {\mathrm{MW}}_{N}=N(\mathrm{\mu g})$$ (5)

where DW_TOT_ and MW_N_ denotes the total DW of the biological replicate and the molecular weight of N, respectively.

Data from the individual isotopologues of L-His and L-Cys peaks were inconclusive and therefore data corresponding to these AAs were excluded.

For the incorporation of N into the AA pool, the ^15^N-amide-L-Gln and ^15^N-amine-L-Gln treatments were omitted from L-Gln calculations since L-Gln^+1^N synthesised *de novo* during the experiment cannot be discriminated from non-metabolised added tracer (^15^N-amide-L-Gln or ^15^N-amine-L-Gln).

The results are presented as mean values ± the standard error of the mean (SE). Significant differences in the experiments were analysed by ANOVA with a Tukey HSD test using JMP Pro software (SAS Institute Inc, USA). A P-value of <0.05 was defined as statistically significant.

## Results

### Positive effect of L-Gln on the growth of PEMs

**Fig 1:**
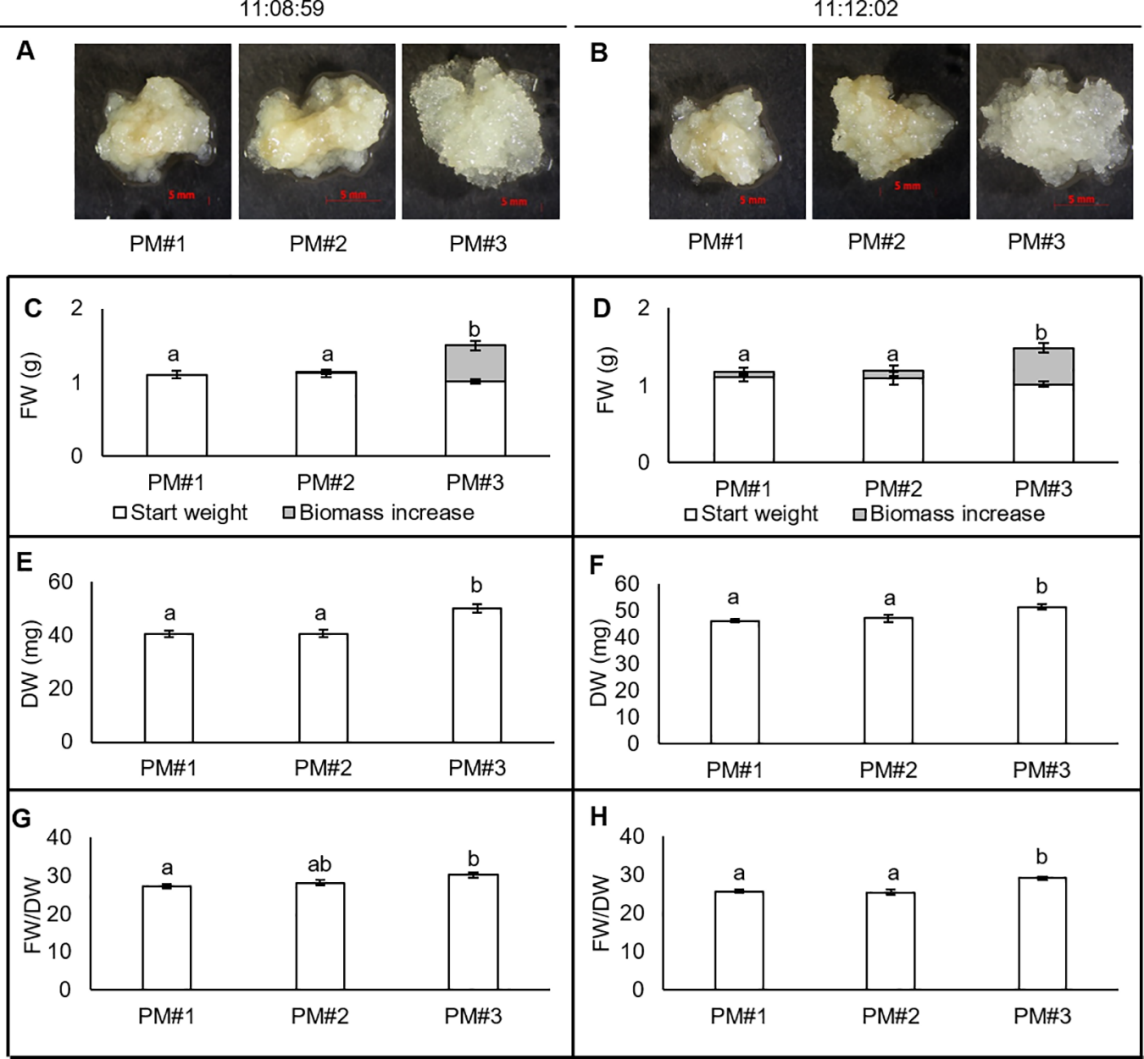
PEM biomass after two weeks of growth on three different experimental media (PM#1–3), for cell lines 11:08:59 and 11:12:02. (A–B) Pictures of the PEMs. (C–D) Biomass. (E–F) Dried biomass. (G–H) Ratio of FW/DW. Each bar represents a mean ± SE; n = 10. Different letters above the bars in respective panels indicate significant differences between the treatments at P<0.05 (Tukey’s test).

We attributed the previously observed positive growth of PEMs on media containing L-Gln to the addition of L-Gln, i.e. to the form of N added rather than to an increase in total N concentration. To verify this hypothesis, we used a combination of three different growth media that allowed the comparison of: i) the same N species with different N concentration (PM#1 vs. PM#2) and (ii) different N species with the same N concentration (PM#2 vs. PM#3). Similar results were obtained for both cell lines, and imaging of each line (Fig 1A and 1B) revealed a satisfactory bumpy-colony structure and whitish translucent colour of the cultures grown on PM#3. However, cultures grown with only inorganic N (PM#1 and PM#2) were unsatisfactory, as evidenced by the smooth colony structure and white-yellow opaque colour. In both cases, with a start weight of 1 g of PEMs (PM#1 and PM#2), two weeks of growth yielded FW values of 1.1 g and 1.2 g for cell lines 11:08:59 and 11:12:02, respectively. In comparison, on PM#3 a FW of 1.5 g was measured for both cell lines (Fig 1C and 1D). Thus, weight increment was 0.1, 0.2 and 0.5 g for PM#1, PM#2 and PM#3, respectively.

With respect to PEMs DW, the two cell lines behaved similarly; weighing ~40 mg, ~40 mg and ~50 mg on PM#1, PM#2 and PM#3, respectively (Fig 1E and 1F). Therefore, the FW and DW of PEMs grown on L-Gln-containing media were higher than PEMs grown on only inorganic sources of N. The FW/DW ratio revealed that PEMs grown on medium PM#3 contained more water than those grown on media with only inorganic N (Fig 1G and 1H).

In the experiment with different concentrations of supplied L-Gln, all treatments with L-Gln displayed significantly higher biomass as compared to PEMs grown on PM#1. Within L-Gln treatments small but insignificant differences were observed (S1 Fig).

In summary, the biomass experiment showed that the addition of L-Gln-N (rather than NH_4_NO_3_-N) resulted in increased growth of PEMs.

### Effect of L-Gln addition on the mass fraction of N, NH_4_^+^ concentration and AA profile of the PEMs

We determined the effect of L-Gln addition on the N status of the PEMs, by evaluating the mass fraction of N, NH_4_^+^ concentration and AA profile of PEMs grown on the different media.

The mass fraction of N was significantly different between all the PM treatments (Table 2). In PEMs grown on PM#3 the mass fraction of N was 4.15 ± 0.08%, PM#2 3.39 ± 0.06%, and PM#1 2.99 ± 0.07%. Thus, PEMs growing in the presence of L-Gln had the highest mass fraction of N compared to the other two treatments with only inorganic N.

**Table 2:** Mass fraction of N in PEMs, from cell line 11:12:02, grown on the three experimental media (PM#1–3).

Treatment	N %
PM#1	2.99 ± 0.07^a^
PM#2	3.39 ± 0.06^b^
PM#3	4.15 ± 0.08^c^

Each value represents a mean ± SE; n = 5 biological replicates. Different letters in respective columns indicate significant differences between the treatments at P<0.05 (Tukey’s test).

Table 3 shows the NH_4_^+^ concentration analysed in the three PM treatments of both cell lines. For both lines, the NH_4_^+^ concentration measured in PEMs grown on PM#2 was significantly higher than that of the other two treatments (PM#1 and PM#3).

**Table 3:** Concentration of NH_4_^+^ per g of DW in PEMs after two weeks of growth on three different experimental media (PM#1–3), for cell lines 11:08:59 and 11:12:02.

	Cell line	
Treatment	11:08:59	11:12:02
PM#1	82.8 ± 2.8^a^	89.6 ± 2.6^a^
PM#2	162.8 ± 7.0^c^	145.0 ± 2.3^b^
PM#3	123.2 ± 9.5^b^	89.0 ± 2.9^a^

Each value represents a mean ± SE; n = 5 biological replicates. Different letters in respective columns indicate significant differences between the treatments at P<0.05 (Tukey’s test).

**Fig 2:**
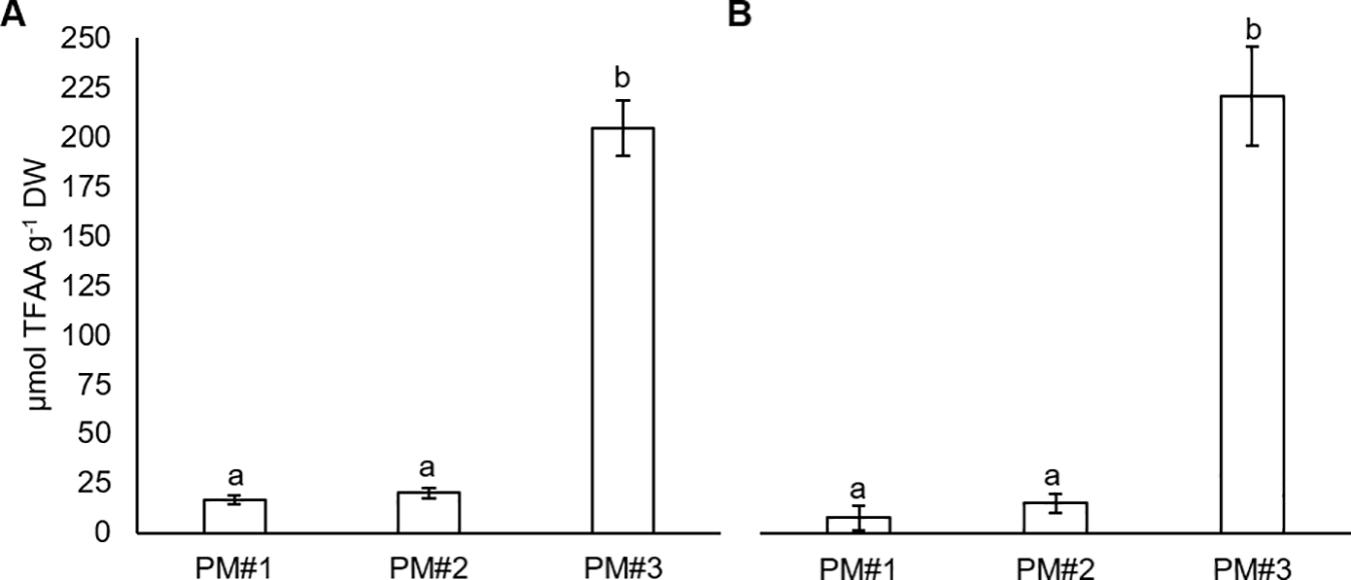
Total free AA pool concentration for PEMs grown on three different experimental media (PM#1–3). (A) Cell lines 11:08:59, (B) 11:12:02. Each bar represents a mean ± SE; n = 5. Different letters above the bars in respective panels indicate significant differences between the treatments at P<0.05 (Tukey’s test).

**Fig 3:**
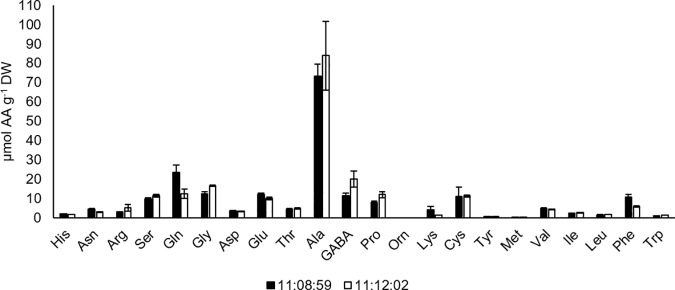
Profile of AA concentrations in PEMs grown on medium PM#3. Cell line 11:08:59 (black bars) and 11:12:02 (white bars). Each bar represents a mean ± SE; n = 5.

Analysis of the total free AA pool revealed negligible differences between the cell lines. In both cases (Fig 2), PEMs grown on PM#3 had a significantly higher concentration of total free AAs than PEMs grown on the other two PM treatments. Cell lines 11:08:59 and 11:12:02 had 10 and 15 times higher total AA concentration, respectively, when grown on PM#3 as compared to growth on PM#1 or PM#2. Analysis of the absolute concentration of each AA measured in PEMs grown on PM#3 (Fig 3) revealed that the concentration of alanine (L-Ala) was remarkably high compared to the other AAs. For PM#1 and PM#2, the concentration of most AAs was very low, or below the detection limit. The results suggest that the supplied L-Gln has a fundamental effect on PEM AA metabolism.

### PEM uptake of organic and inorganic N

The uptake profile of different N sources can provide an overview of the N utilisation in the organism studied. To determine the effect of different media on the N status of the PEMs, N uptake was assessed via reciprocal ^15^N-labelling of the individual forms of N in the medium (PM#3), and subsequent EA-IRMS analysis. In this set-up, the amount of ^15^N detected from each N source will reflect the gross uptake of that source in the presence of all available N sources.

**Fig 4:**
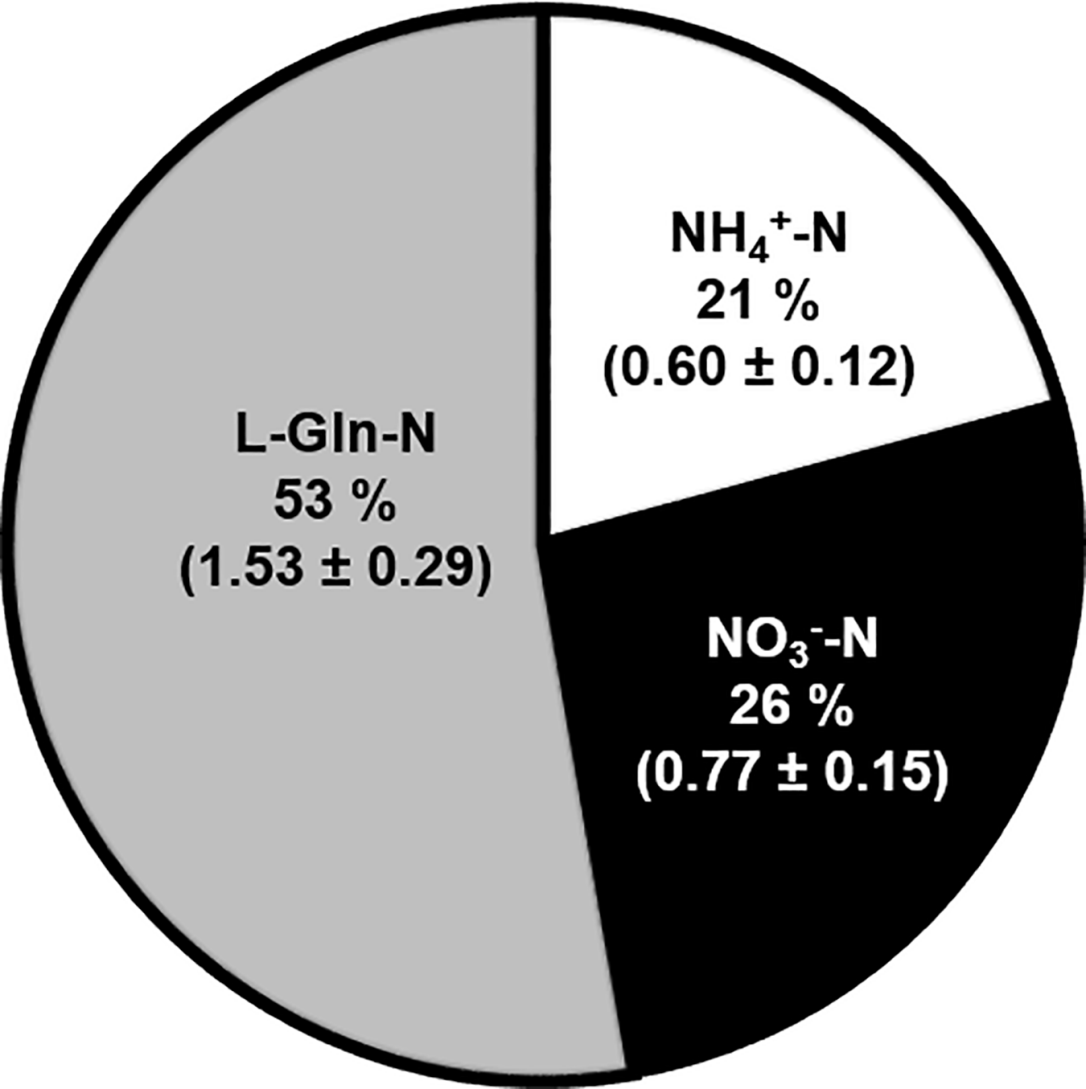
Fraction and amount of N from each N source, NH4^+^, NO3^-^ and L-Gln detected in the PEMs (mean mg N ± SE; n = 10).

The total N concentration of the growth media was 23 mM (8.05 mg plate^-1^). Furthermore, N sources added to these media had N concentrations of 3.7 mM NH_4_-N, 13.1 mM NO_3_-N and 6.2 mM L-Gln-N, representing 16%, 57% and 27% of total N and 1.31, 2.16 and 4.60 mg N plate^-1^. The two-week growth period yielded a total N uptake of 2.9 mg per plate of PEMs. The respective N sources (NH_4_-N, NO_3_-N and L-Gln-N) contributed 0.60 ± 0.12, 0.77 ± 0.15 and 1.53 ± 0.29 mg N g DW^-1^ of N to the PEMs. Therefore, the total N taken up from the media by the PEMs consists of 21% NH_4_^+^-N, 26% NO_3_^-^-N and 53% L-Gln-N (Fig 4). This suggests that the uptake of different N sources is regulated, with PEMs preferring L-Gln and NH_4_^+^ to NO_3_^-^.

### L-Gln and NH_4_^+^—The main contributors to AA N

To stimulate growth, the N taken up must be assimilated into AA metabolism. Therefore, we determined whether the different N sources taken up from the proliferation media were incorporated into plant metabolism by tracing ^15^N-labelled N sources into primary N assimilation, i.e. the free AA pool. PEMs were grown on media (PM#3) amended separately with ^15^N-NO_3_^-^, ^15^N-NH_4_^+^, ^15^amide-L-Gln or ^15^amine-L-Gln. After two weeks, ^15^N was traced to free AAs using LC-MS.

**Fig 5:**
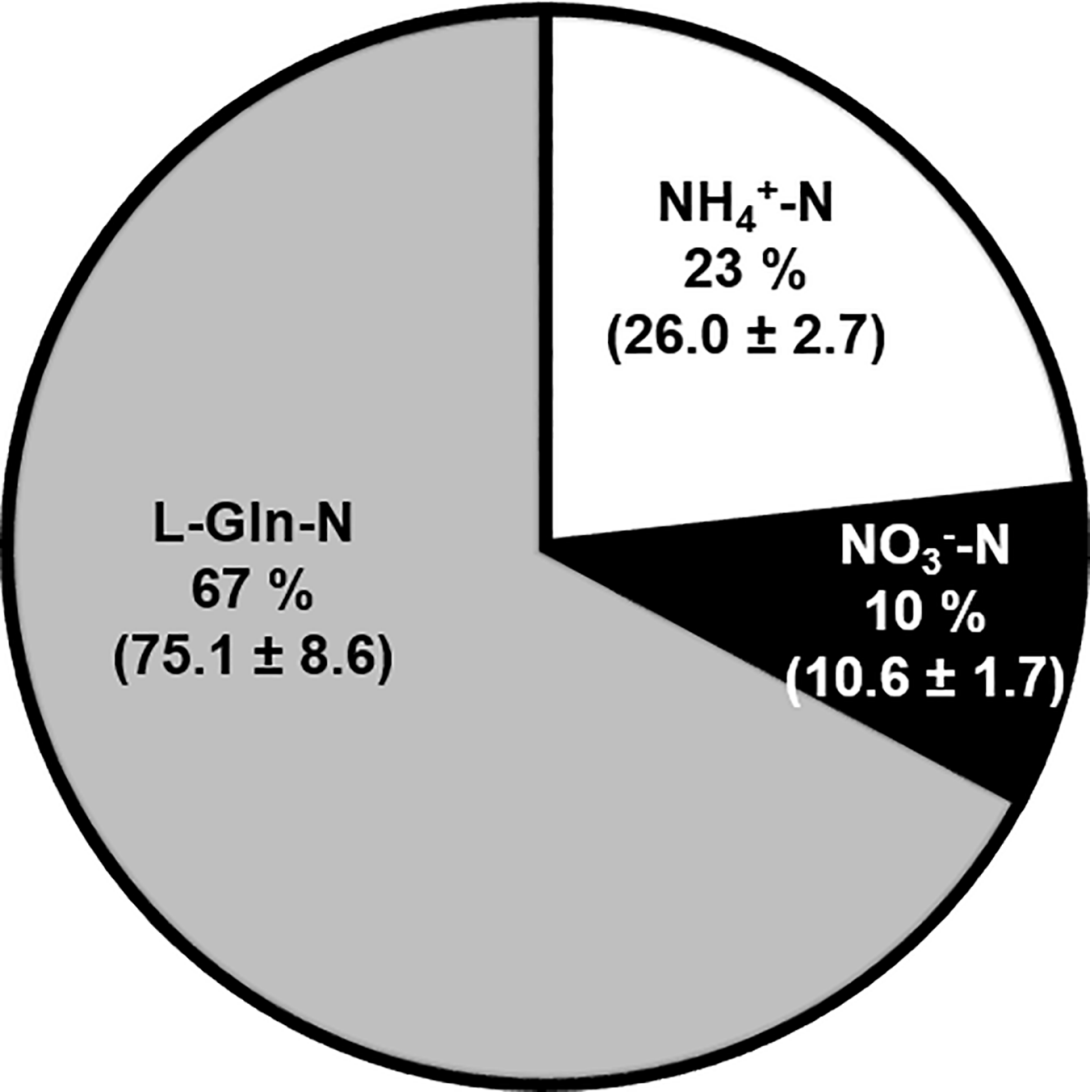
Assimilation of N into the total free AA pool in PEMs grown on PM#3. Fraction and amount of N from each N source, NH_4_^+^, NO_3_^-^ and L-Gln (mean μg N ± SE; n = 9–10).

In this experiment, the analysis was conducted after two weeks of proliferation. The results therefore reflect the incorporation of different N sources into the AA pool over two weeks, rather than the immediate uptake and instantaneous incorporation into AAs or the metabolic pathways leading to subsequent assimilation. The three N-sources provided in the growth medium were all incorporated into AAs (Fig 5). The free AA pool of two-week-old PEMs consisted of 23% NH_4_^+^-N, 10% NO_3_^-^-N and 67% L-Gln-N. Moreover, the analysis revealed that N in individual AAs originated mainly from L-Gln (S2 Fig). Since *de novo* synthesis of L-Gln cannot be distinguished in either the ^15^N-amide-L-Gln or the ^15^N-amine-L-Gln treatments, the amount of N measured in L-Gln is showed separately from the other AAs (S3A Fig). In addition, S3B Fig shows the percentages of NH_4_^+^-N and NO_3_^-^-N, which actually were assimilated and used for synthesising L-Gln *de novo*. The analysis also showed that NO_3_^-^-N was not detectable in L-Phe and L-Ile, and present in only minute amounts in L-Leu, L-Val, L-Orn and GABA (S2 Fig). Thus, after two weeks, the N assimilated into the AA metabolism originated mainly from L-Gln, and then NH_4_^+^ followed by NO_3_^-^.

## Discussion

The yield of plants from the SE process depends on the quantity and quality of PEMs in an embryogenic culture that can respond to the maturation treatment. N is fundamental to plant growth and, hence, crucial for efficient production of healthy PEMs. Therefore, many studies have addressed the influence and role of inorganic and organic N sources during the processing of conifer SE, including Norway spruce. The consensus is that L-Gln or other forms of organic N supplied in the proliferation medium has a positive effect on PEM growth and subsequent somatic embryo development in conifers [44–45, 53–54, 58]. Historically, media composition for *in vitro* applications has been developed by factorial design experiments, generating quantitative or qualitative improvements, but without investigating the physiological consequences of media manipulations. Increasing our knowledge of organic-N nutrition in PEMs may therefore provide information essential for further optimisation of the SE process. To further elucidate the mechanisms governing the observed growth increase induced by organic N, we performed a series of experiments investigating the uptake and utilisation of L-Gln in PEMs.

### Effect of L-Gln on the growth and N status of PEMs

We found, as in previous studies [44–45, 51, 53–54, 58], that L-Gln addition enhances PEM growth, particularly with respect to the fresh weight. Increasing the total N concentration by adding 3.1 mM NH_4_NO_3_ to the base medium did not increase PEM growth. However, as we hypothesised, when 3.1 mM L-Gln was added, the biomass (FW) of PEMs was, on average, 50% higher than that of PEMs grown on NH_4_NO_3_ only (Fig 1C and 1D). Furthermore, the DW of PEMs grown on PM#3 was higher than those of PEMs subjected to the other treatments; DW increased (by 20%) from ~40 mg on PM#1 and PM#2 to ~50 mg on PM#3 (Fig 1E and 1F). Supplying L-Gln at half or double concentrations (PM#4 and PM#5), did not significantly change PEM growth as compared to PM#3 (S1 Fig). However, further investigation of the effects different L-Gln concentrations have on Norway spruce PEM metabolism, and further optimisation of media composition for PEM growth in different applications, could have merit. The results from this study supports our hypothesis that the observed growth increase was mediated by the N species (i.e. L-Gln), and not by addition of increased amounts of N. Furthermore, the visual appearance of the calluses (Fig 1A and 1B) indicates that the PEMs grown on PM#3 were healthier than those grown on the other two media. Addition of L-Gln also had a pronounced effect on the N status of the PEMs. The significantly lower tissue mass fraction of N (Table 2) and total free AA pool concentration (Fig 2) in PEMs grown on PM#1 and PM#2, in comparison with PM#3, suggests that media without L-Gln, is inadequate for maintaining a functioning N-metabolism of PEMs. This is further emphasised by the finding that although PM#2 media contained more NH_4_NO_3_ than PM#1 media and that PM#2 PEMs had a higher tissue mass fraction of N than on PM#1 (Table 2), growth was still not improved.

### PEM uptake of organic and inorganic N

Nutrient utilisation depends on complex and intricately regulated uptake systems that allow nutrient transport over inter- and intracellular membranes. With respect to uptake, different species have different affinities for N; for example, preferential uptake of NH_4_^+^ and AAs, compared with that of NO_3_^-^, has been reported for conifer seedlings [19–28, 59–60]. However, the uptake preference does not necessarily reflect impact on growth, i.e. the form of N that would yield optimum growth. The increased growth of PEMs on L-Gln-containing media may nevertheless have resulted from a N-source preference, which (in this work) is referred to as the differential uptake of the supplied N sources. We hypothesised that PEM cells regulate the uptake of different N sources, with a relatively higher uptake of L-Gln than inorganic N. This was verified by growing PEMs on a medium (PM#3) with the reciprocal ^15^N-label of each supplied N-source. Thus, preference for a N source would lead to a discrepancy between the relative N composition of PEMs and the N composition of the growth medium. In this study, all three N sources were taken up by the PEMs. However, the fraction of each N source taken up differed from the respective fractions provided in the medium (Fig 4). This suggests that the uptake of N was regulated, with PEMs preferring L-Gln and NH_4_^+^ to NO_3_^-^.

### L-Gln and NH_4_^+^—The main contributors to AA N

Assimilation of inorganic N requires a supply of C-skeletons, indicative of the close correlation between C- and N-metabolism [61–64]. In a non-photosynthetic tissue culture system, C-skeletons and energy are commonly provided as sucrose in the media. Energy and the required C-backbone must therefore be produced from glucose (through glycolysis) and the tricarboxylic acid cycle, prior to the assimilation of inorganic N [61, 63]. The addition of L-Gln to the culture medium provides the tissue culture with already assimilated C and N, thereby saving energy. This may be significant during the heterotrophic state where the PEMs are unable to perform photosynthesis. Moreover, the C-backbone of L-Gln may be important for PEM metabolism, i.e. as a respiratory substrate or for the synthesis of metabolites. However, isotopic ^13^C labelling was not included in this study and, hence, this possibility needs to be addressed in future work.

In this work, ^15^N-labelled N sources were traced into primary N assimilation, and it was found that N originating from all N sources occurred in almost all AAs analysed (S2 Fig). Although NO_3_^-^-derived N comprised 26% of the total N uptake, NO_3_^-^-N accounted for only 10% of the free AA pool (Fig 5). This may be the result of NO_3_^-^ remaining in the intercellular space, low capacity for nitrate and nitrite reduction, and/or storage of NO_3_^-^ in vacuole [39, 64]. Alternatively, low incorporation of NO_3_^-^ could indicate low GS activity. However, this can be ruled out, since the free AA pool contained 23% NH_4_-N, indicative of functional GS. Most notably in our study, 67% of the N in the free AA pool originated from either the L-Gln amide or amine group. With respect to NH_4_^+^ and NO_3_^-^, these findings concur with those of Joy with colleagues [56] who found that N from ^15^N-NH_4_ and ^15^N-NO_3_ occurred first in the amide group of L-Gln, indicative of a functional GS/GOGAT system in white spruce SE. Similar to the findings in this study, Joy with colleagues [56] also observed that, NH_4_-N was readily incorporated and NO_3_^-^-N was more slowly incorporated (and in smaller amounts), into L-Gln.

Given the relatively high fraction of NO_3_^-^-N in the media versus that present in the PEMs, and the low amount of NO_3_^-^-N assimilated into AAs, it could be argued that only a minor part of the total N content in the media is available to PEMs. Therefore, in the absence of L-Gln, PEMs must rely on NH_4_^+^ for N, which suggests that PEMs grown on such media could experience N-limitation. On the other hand, increased growth did not occur on PM#2 medium that contained almost double the amount of NH_4_^+^-N present in PM#1. This suggests that L-Gln in PEMs functions in other capacities in addition to being a source of N. At this stage, we can only speculate about the mechanisms through which L-Gln alleviates these metabolic constraints. We conclude that all N sources taken up were assimilated, albeit to different extents. In contrast to the relatively small amount of NO_3_^-^-N incorporated into AAs in PEMs, NH_4_^+^-N was readily incorporated into AAs. Most importantly, the results reveal the necessity of added L-Gln, which contributed to two-thirds of the total free AA pool.

In this study, analysis of the total free AA pool of PEMs grown in the presence of L-Gln revealed a striking high amount of assimilated N in L-Ala, which represented ~66% of the N in total free AA pool in PEMs grown on PM#3. This suggests that the mechanism governing AA metabolism of PEMs grown in the presence of L-Gln differs from that governing the metabolism of PEMs where inorganic N is the only source of N. As in the present study, Joy with colleagues [56] reported a high L-Ala concentration for embryogenic white spruce cultures grown on a solidified medium. High concentration of L-Ala has also been attributed to anaerobic stress in *Arabidopsis thaliana* [65–66], barley (*Hordeum vulgare* L. cv Himalaya) [67], *Medicago truncatula* [68], rice and poplar [66]. Miyashita with colleagues [65] proposed that L-Ala production might be a route through which plants preserve N and C during hypoxia. Limami with colleagues [69] hypothesised that under low-oxygen stresses, C is stored in the form of L-Ala via an alanine aminotransferase/glutamate dehydrogenase cycle. L-Ala is synthesised from pyruvate and L-Glu via alanine aminotransferase. In the reverse reaction, from L-Ala and 2-oxoglutarate, pyruvate is funnelled to the Krebs cycle, while the oxidative deamination of L-Glu (by GDH) restores 2-oxoglutarate that maintains the cycle and generates NADH. However, it remains unclear whether plants simply store L-Ala until favourable growth conditions are restored or if the AA plays an additional role in ensuring anaerobic stress tolerance. Another hypothesis suggests that L-Ala might be a way of regulating the production of acetaldehyde, an intermediary in the ethanol biosynthetic pathway, which is harmful to plants. By competing for pyruvate, L-Ala synthesis limits the production of acetaldehyde and saves C-skeletons [68]. The observed high L-Ala concentration of the PEMs may also have resulted from the so-called GABA shunt. This shunt is a metabolic pathway that starts with L-Glu (from either the amination of 2-oxoglutarate or deamination of L-Gln) and generates GABA through the action of glutamate decarboxylase. GABA transaminase catalyses the reaction of GABA and pyruvate to L-Ala and succinic semialdehyde. The succinic semialdehyde is subsequently dehydrogenated by succinic semialdehyde dehydrogenase, generating NADH [70–71]. However, which of the pathways that yields the high L-Ala concentrations have to be elucidated in future experiments. Similarly, the mechanisms underlying the stress that seems to render L-Gln essential for PEM N metabolism needs to be further investigated.

In conclusion, this study shows that, compared to NH_4_^+^ and NO_3_^-^, L-Gln had a more positive effect on the growth of PEMs. PEMs appeared to have a controlled, regulated uptake of the different N sources, with a preference for L-Gln and NH_4_^+^ to NO_3_^-^. All three N-sources were assimilated into AA metabolism; of the N occurring in the free AA pool, 67%, 23% and 10% originated from L-Gln-N, NH_4_^+^-N and NO_3_^-^-N respectively. This highlights the importance of determining the actual uptake in relation to the media composition since the most abundant N-source in the media, NO_3_^-^, is the least abundant after assimilation. The addition of L-Gln appears essential for sustaining cell metabolism under the prevailing conditions. However, the high level of L-Ala in PEMs grown in the presence of L-Gln suggests that these PEMs still experience some form of stress. It is important to emphasise, that any metabolic constraints in the *in vitro* phase will probably also affect downstream processes studied in said systems, e.g. embryo development. Also, adding new compounds without understanding the physiological consequences might cause problems further down the optimisations chain. Hence, the results presented here motivate additional studies of N nutrition, and perhaps not only for Norway spruce SE. Given the widespread use of organic N in plant *in vitro* culture, the findings in this study, especially with respect to the addition of L-Gln may be relevant for many plant *in vitro* applications.

## Supporting information

S1 Table Experimental proliferation media compositions with different concentrations of L-Gln. (DOCX)

S1 Text Method used for targeted AA analysis using LC-MS. (DOCX)

S1 Fig PEM biomass after two weeks of growth on four different experimental media (PM#1; 3–5), for cell line 11:12:02. (A) Biomass. (B) Dried biomass (C) Ratio of FW/DW. Each bar represents a mean ± SE; n = 7. Different letters above the bars in respective panels indicate significant differences between the treatments at P<0.05 (Tukey’s test).
(TIF)

S2 Fig N origin in the free pool of individual AAs. Fraction from each N source, NH_4_^+^, NO_3_^-^ and L-Gln (mean μg N ± SE; n = 9–10).
(TIF)

S3 Fig N origin in L-Gln measured in the free pool of AAs. (A) Fraction from each N source, NH_4_^+^, NO_3_^-^, amide-L-Gln and amine-L-Gln. (B) Fraction from the inorganic N sources. Each bar represents a mean ± SE; n = 9–10.
(TIF)
