# Correction: Nitrogen uptake and assimilation in proliferating embryogenic cultures of Norway spruce—Investigating the specific role of glutamine

**DOI:** 10.1371/journal.pone.0191208

**Published:** 2018-01-19

**Authors:** Johanna Carlsson, Henrik Svennerstam, Thomas Moritz, Ulrika Egertsdotter, Ulrika Ganeteg

The authors have stated that there was an error in the method used to subtract natural abundance of amino acid isotopologues. The authors have now re-calculated the results.

There are errors in the “Calculations and statistical treatment of data” section in the Materials and methods. The authors have also added three new equations. Please see the corrected “Calculations and statistical treatment of data” section here:

The amounts of N derived from the different N sources are reported in units of mg N in the tissue, calculated using the formula:

$$\left(\left(atom\%\;{}^{15}N_{LS}-atom\%\;{}^{15}N_{NAT}\right)/100)\times (\left({N\%}_{TOT}\right)/100)\times DW(mg)\times MF=N_{LS}\;(mg\right)$$ (1)

where the subscript “LS” denotes labelled sample, “NAT” natural abundance (here 0.3663% is used for all samples) and MF denotes Multiplication Factor. The contribution of each N form is given as % of total N uptake during the labelling experiment.

The amount of N (μg) assimilated from the different N sources into AAs in the free AA pool was calculated in a stepwise manner.

Initially, the natural abundance is subtracted from labelled samples. This is done by using correction factors calculated from non-labelled control samples (NLS) for each individual AA and isotopologue (m^+1^ and m^+2^):

$$Area\;{AA}_{NLS}^{+n}/Area\;{AA}_{NLS}^{+0}={fraction}_{NLS}^{+n}$$ (2)

where n denotes the increase in molecular mass as compared to the mono-isotopic form.

To calculate AA excess peak areas in labelled samples, the natural abundance of ^15^N has to be subtracted using the correction factors calculated in Eq 2:

$$Area\;{AA}_{LS}^{+1}-(Area\;{AA}_{LS}^{+0}\times {fraction}_{NLS}^{+1})=Area\;{AA}_{LS}^{+1}\;excess$$ (3)

$$Area\;{AA}_{LS}^{+2}-(Area\;{AA}_{LS}^{+0}\times {fraction}_{NLS}^{+2})-(\;Area\;{AA}_{LS}^{+1}\;excess\;\times {fraction}_{NLS}^{+1})=Area\;{AA}_{LS}^{+2}\;excess$$ (4)

The corrected AA peak areas are then transformed to fractions of total AA peak area:

$${Area\;AA}_{LS}^{N}\;/\left({Area\;AA}_{LS}^{0}+{Area\;AA}_{LS}^{+1}+{Area\;AA}_{LS}^{+2}\right)=Fraction\;{AA}_{LS}^{n}$$ (5)

The concentration (μmol/g DW) of each isotopologue [AA^n^] was calculated as:

$$Fraction\;{AA}_{LS}^{n}\times {\left[AA\right]}_{LS}=[{AA}^{n}]$$ (6)

where [AA]_LS_ represents the concentration of the AA in the sample determined by UPLC- analysis.

The concentration of N (μmol /g DW) derived from the labelled N source [N]_LS_ was calculated as:

$$[{AA}^{+1}]+([{AA}^{+2}]\times 2)={\left[N\right]}_{LS}$$ (7)

Lastly, the absolute amount of N (μg) derived from the labelled N source in each biological replicate was calculated as:

$${\left[N\right]}_{LS}\times {DW}_{TOT}\times {MW}_{N}=N(\mu g)$$ (8)

where DW_TOT_ and MW_N_ denotes the total DW of the biological replicate and the molecular weight of N, respectively.

Data from the individual isotopologues of L-His and L-Cys peaks were inconclusive and therefore data corresponding to these AAs were excluded.

For the incorporation of N into the AA pool, the ^15^N-amide-L-Gln and ^15^N-amine-L-Gln treatments were omitted from L-Gln calculations since L-Gln^+1^N synthesised *de novo* during the experiment cannot be discriminated from non-metabolised added tracer (^15^N-amide-L-Gln or 1^5^N-amine-L-Gln).

The results are presented as mean values ± the standard error of the mean (SE). Significant differences in the experiments were analysed by ANOVA with a Tukey HSD test using JMP Pro software (SAS Institute Inc, USA). A P-value of <0.05 was defined as statistically significant.

In Fig 5, the values and percentages of N originating from each N source are incorrect. Please see the corrected Fig 5 here.

**Fig 5:**
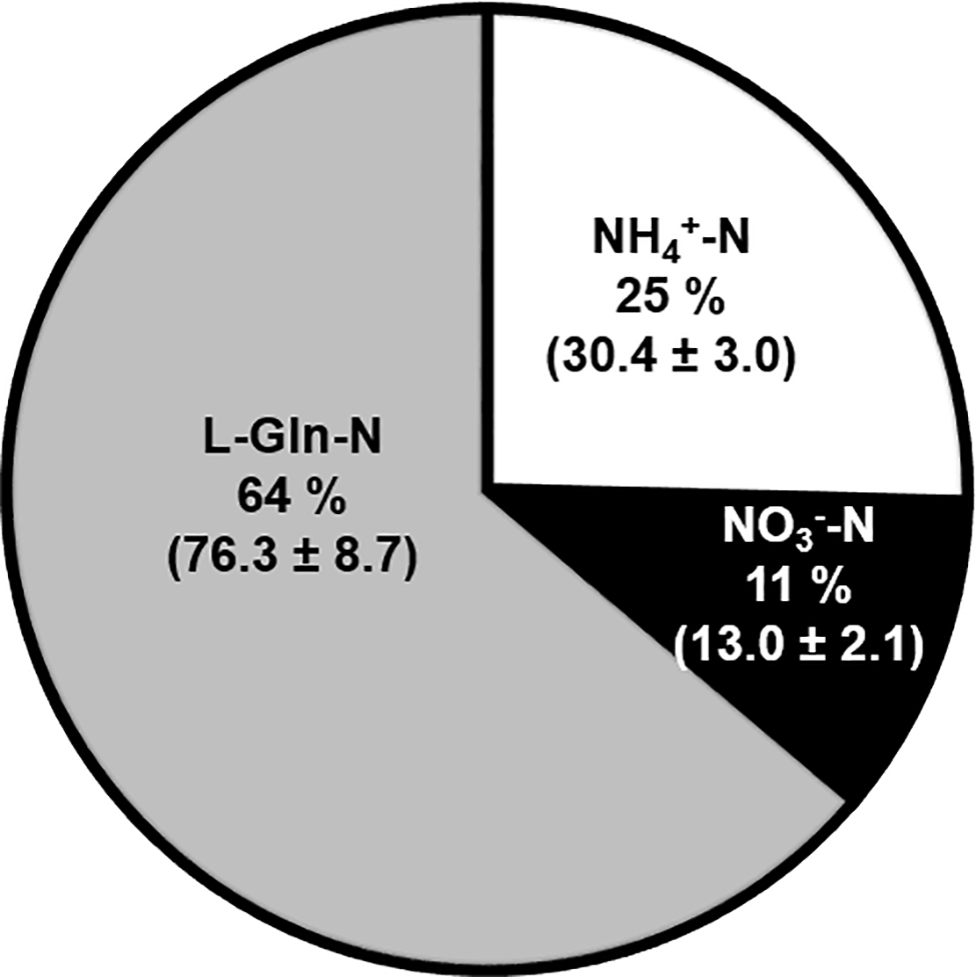
Assimilation of N into the total free AA pool in PEMs grown on PM#3. Fraction and amount of N from each N source, NH_4_^+^, NO_3_^-^ and L-Gln (mean μg N ± SE; n = 9–10).

S2 Fig and S3 Fig have been updated after the re-calculation. Please see the corrected S2 Fig and S3 Fig below.

## Supporting information

S2 Fig N origin in the free pool of individual AAs. Fraction from each N source, NH_4_^+^, NO_3_^-^ and L-Gln (mean μg N ± SE; n = 9–10).
(TIF)

S3 Fig N origin in L-Gln measured in the free pool of AAs. (A) Fraction from each N source, NH_4_^+^, NO_3_^-^, amide-L-Gln and amine-L-Gln. (B) Fraction from the inorganic N sources. Each bar represents a mean ± SE; n = 9–10.
(TIF)
