# Supplementary material for: Nitrogen uptake and assimilation in proliferating embryogenic cultures of Norway spruce—Investigating the specific role of glutamine
Source: PLoS One. 2017 Aug 24;12(8):e0181785. doi: 10.1371/journal.pone.0181785 (PMC5570297; doi:10.1371/journal.pone.0181785)
Supplement: S1 Table — (DOCX) [file pone.0181785.s001.docx]

**S1 Table.**  **Experimental proliferation media compositions with different concentrations of L-Gln.**

|  |  | Total N | NH_4_^+^-N | | NO_3_^-^-N | | L-Gln-N | | |
| --- | --- | --- | --- | --- | --- | --- | --- | --- | --- |
| Medium | Treatment | mM | mM | % | mM | % | mM | % | |
| ½-LP | PM#1 | 16.8 | 3.7 | 22.2 | 13.1 | 77.8 | - | - |  |
| ½-LP + 1.5 mM L-Gln | PM#4 | 19.9 | 3.7 | 18.6 | 13.1 | 65.8 | 3.1 | 15.6 |  |
| ½-LP + 3.1 mM L-Gln | PM#3 | 23.0 | 3.7 | 16.3 | 13.1 | 57.0 | 6.2 | 26.7 |  |
| ½-LP + 6.2 mM L-Gln | PM#5 | 29.4 | 3.7 | 12.6 | 13.1 | 44.6 | 12.4 | 42.2 |  |

Experimental media, corresponding concentrations of total N and the N concentration of each N source (NH_4_^+^, NO_3_^-^ and L-Gln) provided in the media.
