# Supplementary material for: Nitrogen uptake and assimilation in proliferating embryogenic cultures of Norway spruce—Investigating the specific role of glutamine
Source: PLoS One. 2017 Aug 24;12(8):e0181785. doi: 10.1371/journal.pone.0181785 (PMC5570297; doi:10.1371/journal.pone.0181785)
Supplement: S1 Text — (DOCX) [file pone.0181785.s002.docx]

**S1 Text. Targeted AA analysis using LC-MS.**

During the LC-MS analysis, the sample extracts were re-suspended in 20 µL of 20 mM HCl. Sixty µL of Waters AccQ•Tag™ Ultra borate buffer and 20 µL of freshly prepared Waters AccQ•Tag™ derivatisation solution was added to each vial and the samples were directly vortex-mixed for 10 s. The samples were incubated for 30 min at room temperature and for 10 min at 55 °C. A ten-point calibration curve ranging from 0.05 to 50 pmol (on the column) was prepared in parallel with the samples and analysed in triplicates. Norvaline, which was used as the internal standard, was maintained at 2.5 pmol on the column of the calibration curve. The samples were analysed via LC-MS. The derivatised AAs were separated using a 1290 Infinitely system (Agilent Technologies, Waldbronn, Germany) consisting of a G4220A binary pump, G1316C thermostated column compartment and G4226A autosampler with G1330B autosampler thermostat. A 2 µL aliquot of the sample was injected onto a 2.1 × 100 mm, 1.7 µM HPLC C18-column (Kinetex, Phenomenex) held at 50 °C in a column oven. The gradient elution buffers were referred to as A (H_2_O, 0.1% formic acid) and B (acetonitrile, 0.1% formic acid), and a flow-rate of 500 µL min^-1^ was employed. Mass spectrometry-grade formic acid and HPLC-grade acetonitrile were purchased from Sigma-Aldrich (St Louis, MO, USA) and Fisher Scientific (Fair Lawn, NJ, USA), respectively. The initial condition (0.1% B) was maintained for 0.54 min. From 0.54 to 5.5 min, the B eluent was linearly increased from 0.1% to 9.1%. After 5.5 min, B was increased linearly, reaching 21.2% at 7.7 min. The percentage of B was further increased to 59.6% from 7.7 min to 8.5 min and held at this value for 0.5 min. The highly non-polar compounds were eluted by rapidly increasing the proportion of B to 80% within 9.5 min and holding at this value for 0.5 min. From 10 to 10.5 min, the initial condition (0.1% B) of the column was restored, and the column was equilibrated for 4.5 min before injecting the subsequent sample. The AAs were detected with an Agilent 6550 QTOF mass spectrometer equipped with a jet-stream electrospray source operating in positive mode, under the following conditions: capillary voltage: 4 kV, jet-stream gas temperature and gas flow: 150 °C and 16 L min^-1^, respectively, sheath gas temperature and gas flow: 350 °C and 11 L min^-1^, respectively, nebuliser pressure: 35 psi, fragmentor voltage: 380 V, collision energy: 0 V, mass range of the TOF: 70–1700 m/z, scan rate: 4 scans s^-1^. Purine (2 µM) and HP-0921 (Hexakis(1H, 1H, 3H-tetrafluoropropoxy)phosphazine) (2.5 µM) (both from Agilent Technologies, Santa Clara, CA, USA) were used as reference masses and infused with an isocratic pump (flow rate: 19 µL min^-1^).
