# Supplementary figures and images for: Nitrogen uptake and assimilation in proliferating embryogenic cultures of Norway spruce—Investigating the specific role of glutamine
Source: PLoS One. 2017 Aug 24;12(8):e0181785. doi: 10.1371/journal.pone.0181785 (PMC5570297; doi:10.1371/journal.pone.0181785)

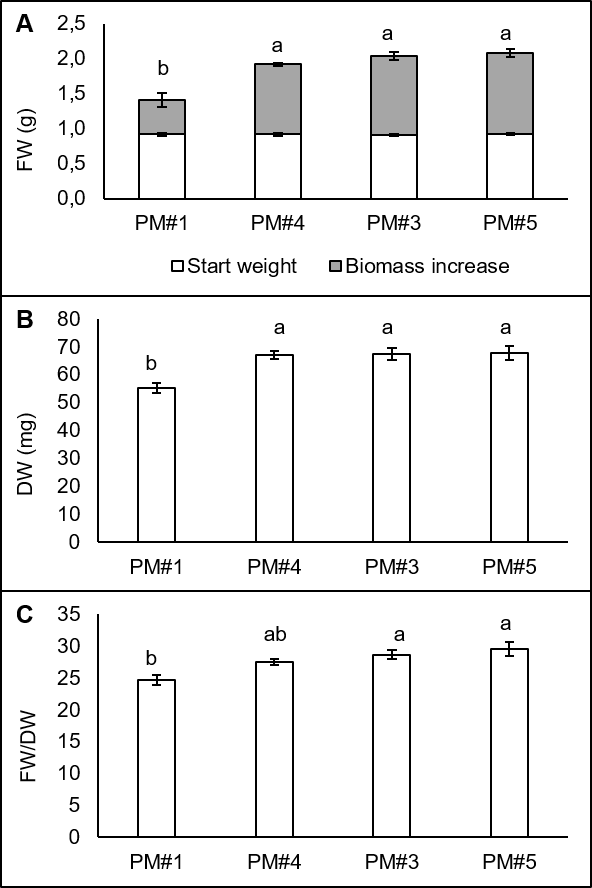

Supplement: S1 Fig — (A) Biomass. (B) Dried biomass (C) Ratio of FW/DW. Each bar represents a mean ± SE; n = 7. Different letters above the bars in respective panels indicate significant differences between the treatments at P<0.05 (Tukey’s test). (TIF) [file pone.0181785.s003.tif]

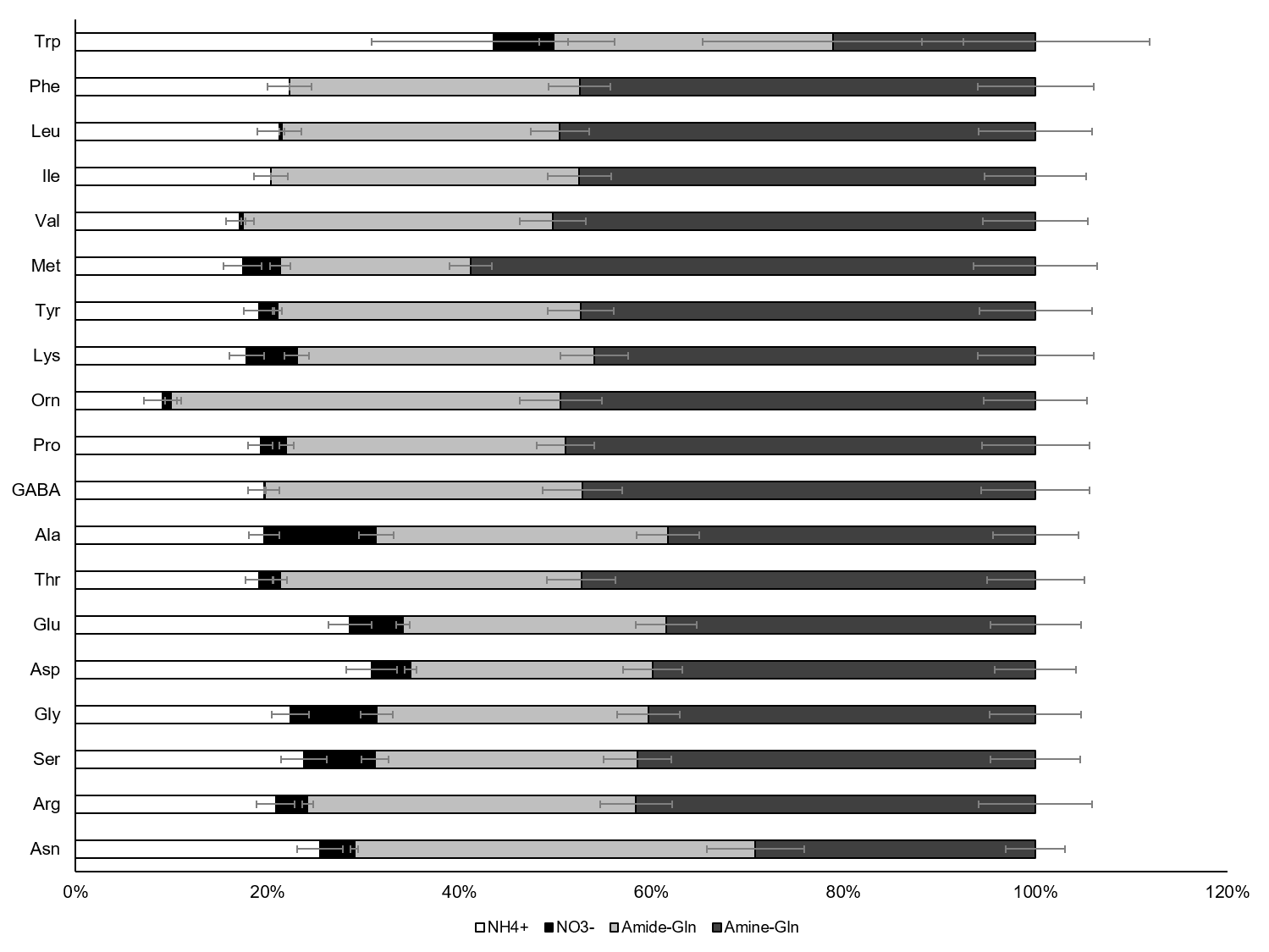

Supplement: S2 Fig — Fraction from each N source, NH4+, NO3- and L-Gln (mean μg N ± SE; n = 9–10). (TIF) [file pone.0181785.s004.tif]

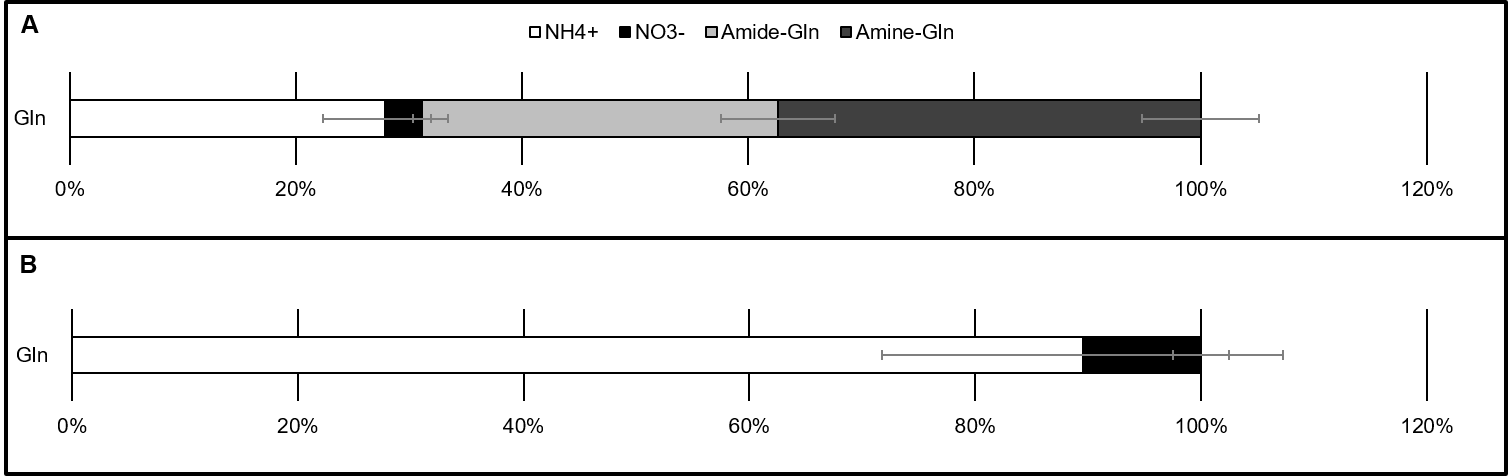

Supplement: S3 Fig — (A) Fraction from each N source, NH4+, NO3-, amide-L-Gln and amine-L-Gln. (B) Fraction from the inorganic N sources. Each bar represents a mean ± SE; n = 9–10. (TIF) [file pone.0181785.s005.tif]
