# Supplementary figures and images for: Correction: Nitrogen uptake and assimilation in proliferating embryogenic cultures of Norway spruce—Investigating the specific role of glutamine
Source: PLoS One. 2018 Jan 19;13(1):e0191208. doi: 10.1371/journal.pone.0191208 (PMC5774748; doi:10.1371/journal.pone.0191208)

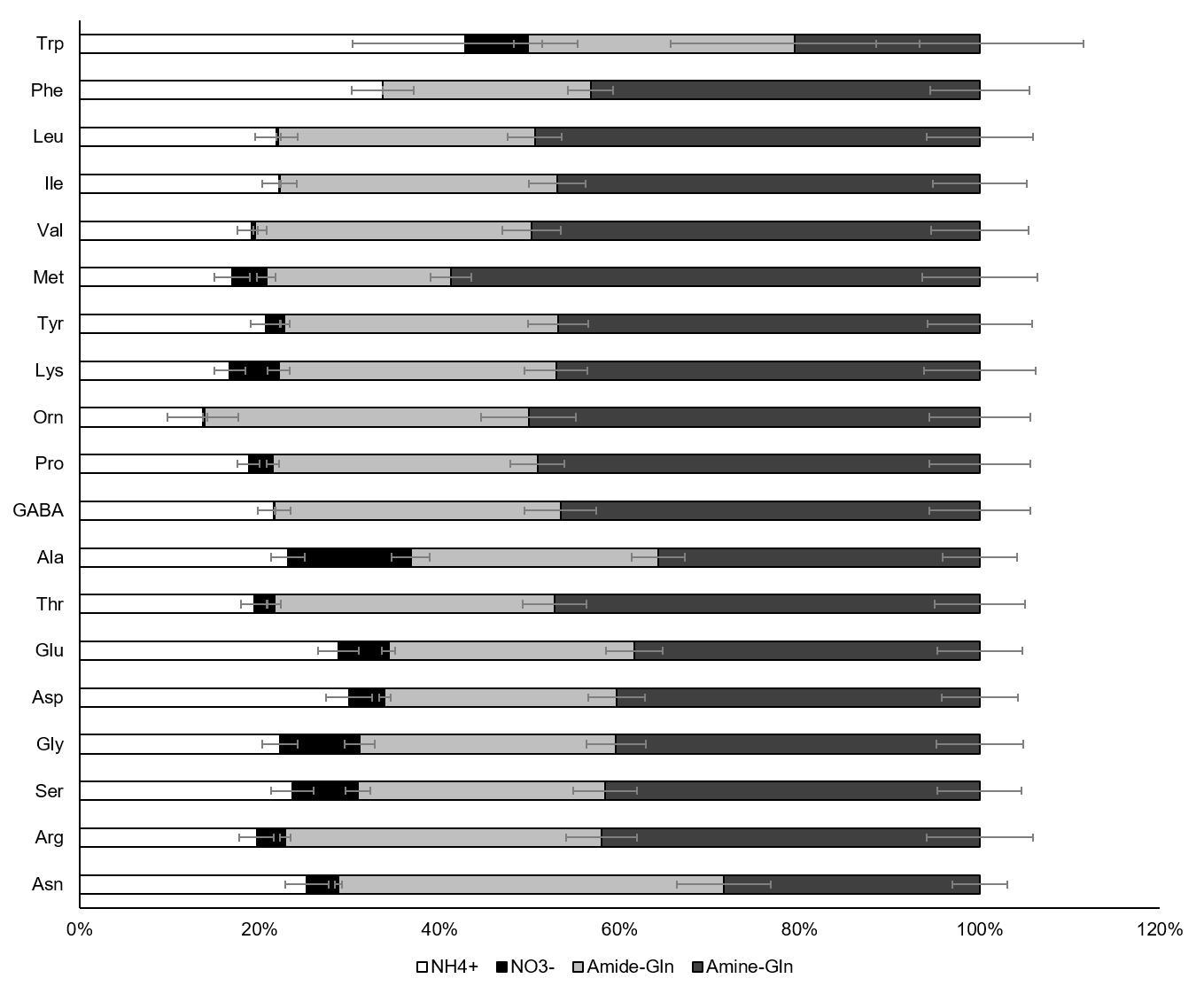

Supplement: S2 Fig — Fraction from each N source, NH4+, NO3- and L-Gln (mean μg N ± SE; n = 9–10). (TIF) [file pone.0191208.s001.tif]

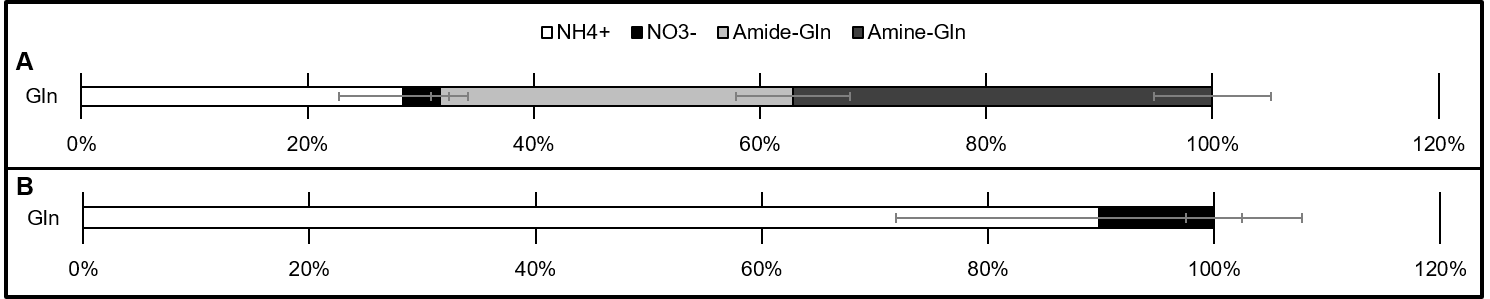

Supplement: S3 Fig — (A) Fraction from each N source, NH4+, NO3-, amide-L-Gln and amine-L-Gln. (B) Fraction from the inorganic N sources. Each bar represents a mean ± SE; n = 9–10. (TIF) [file pone.0191208.s002.tif]
